# Myeloid-specific SIRT1 deletion exacerbates airway inflammatory response in a mouse model of allergic asthma

**DOI:** 10.18632/aging.203104

**Published:** 2021-06-07

**Authors:** Tianwen Lai, Guomei Su, Dong Wu, Ziyu Chen, Yujuan Chen, Huajuan Yi, Yun Gao, Cuifen Chen, Man Zeng, Min Chen, Dongming Li, Bin Wu

**Affiliations:** 1 Department of Respiratory and Critical Care Medicine, Affiliated Hospital of Guangdong Medical University, Zhanjiang 524001, China

**Keywords:** SIRT1, asthma, BMDM, macrophage

## Abstract

Sirtuin 1 (SIRT1) is a class III histone deacetylase that exerts an anti-inflammatory effect in airway diseases. Activated macrophages play an important role in asthma. However, the roles of SIRT1 on allergic airway inflammation in macrophages remain largely unexplored. In this study, we aimed to determine the roles of SIRT1 on allergic airway inflammation in macrophages. The effect of myeloid-specific SIRT1 deletion (*Sirt1^fl/fl^*-*LysMcre*) on airway inflammation was assessed by using *in vivo* models of asthma following allergen exposure and *in vitro* culture of primary bone marrow–derived macrophages (BMDMs) exposed to house dust mite (HDM). We observed that *Sirt1^fl/fl^*-*LysMcre* mice substantially enhanced airway inflammation and mucus production in response to allergen exposure. Expression of chemokine ligand (CXCL) 2, interleukin (IL)-1β, and tumor necrosis factor (TNF)-α were reduced in BMDMs with myeloid-specific deletion of *Sirt1* after stimulation of HDM. Moreover, SIRT1 suppressed the inflammatory cytokines expression in BMDMs partially via the ERK/p38 MAPK pathways. Our study demonstrated that SIRT1 suppresses the allergic airway inflammation in macrophages, and suggested that activation of SIRT1 in macrophages may represent therapeutic strategy for asthma.

## INTRODUCTION

Bronchial asthma is characterized by airway hyperresponsiveness (AHR), variable airflow limitation, and airway remodeling [1]. Currently, there is no effective therapy for this disease [1, 2]. Therefore, it is critical to determine the molecular mechanisms of asthma.

Although the airway epithelial cells serve as a first defending barrier of defense against invading pathogens in asthma, recent studies have indicated that macrophages play an important role in fighting against pathogenic invasion [3, 4]. Bang et al. demonstrated that allergic airway inflammation was significantly ameliorated when unsensitized alveolar macrophages (AMs) were adoptively transferred to macrophage-depleted sensitized mice [5]. However, the underlying mechanisms of AMs in regulation of allergic airway inflammatory remain unknown.

Sirtuin 1 (SIRT1) plays an important role in regulating many pathophysiological processes, such as inflammation, autoimmunity, and apoptosis [6]. Our previous study has shown that serum SIRT1 levels in patients with asthma were positively associated serum IgE levels [7]. Ichikawa et al. found that SIRT1 activator suppressed inflammatory cell infiltration and cytokine production in asthmatic mice [8]. Kardan et al. discovered that SIRT1 protein levels were decreased in patients with severe asthma [4]. Although AMs are the first responders to foreign pathogens in the airway and secrete proinflammatory response upon exposure to outside invaders, the possible role of SIRT1 in airway inflammation of macrophages following allergen exposure remains unclear.

Here, using an experimental model of asthma, we sought to determine the underlying mechanisms of SIRT1 in the regulation of allergic airway inflammatory responses in macrophages using an experimental model of asthma. Our findings revealed that ERK/p38 MAPK pathways being the underlying mechanism by which SIRT1 regulates the response of macrophages upon allergen exposure to regulate airway inflammation and mucus production.

## RESULTS

### SIRT1 expression is decreased in macrophages following allergen exposure

**Figure 1:**
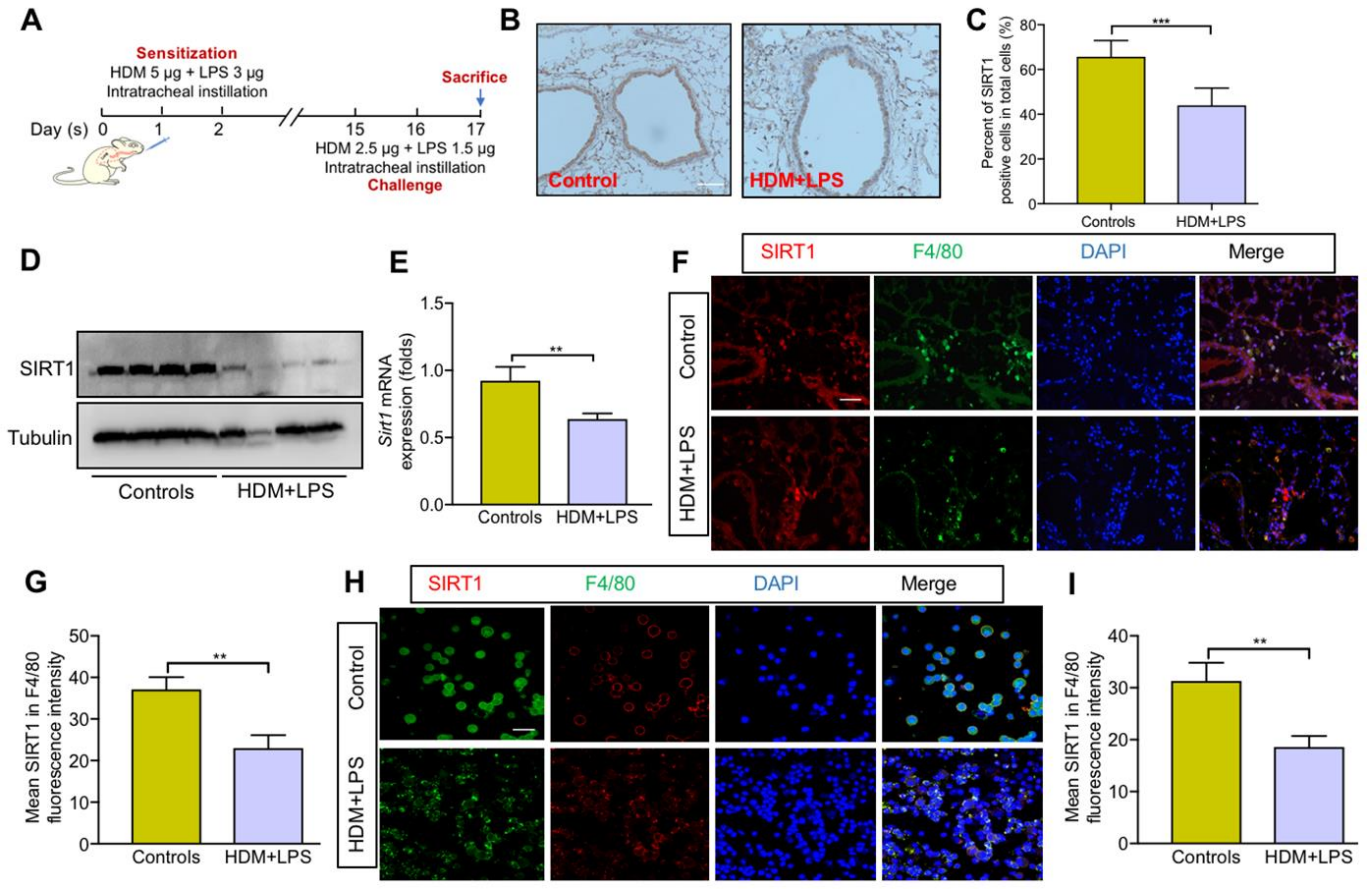
**SIRT1 expression is decreased in macrophages following allergen exposure.** (**A**) Schematic map of allergic asthma model; (**B**) Representative IHC images of SIRT1 in lung tissue of mice (scale bar, 100 μm); (**C**) Semiquantification of IHC was done using Image Pro 6.1 software; (**D**, **E**) SIRT1 expression in lung tissues of *Sirt1^fl/fl^* mice were assessed using Western blot and RT-PCR analysis; Representative immunofluorescence images of SIRT1 expression in alveolar macrophages are revealed using F4/80 in lung tissues (**F**) and BALF (**H**) of mice (Scale bar, 100 μm and 20 μm, respectively); (**G**, **I**) Semiquantification of immunofluorescence images was done using Image Pro 6.1 software. Data are presented as Mean ± SEM of three independent experiments (n = 5-8 for each group). **P<0.01 and ***P<0.001.

We first assessed SIRT1expression in the lung tissue of mice. Allergic asthma model was established in of *Sirt1^fl/fl^* mice, as described in Figure 1A. The results IHC staining revealed that the expression of SIRT1 was significantly decreased in lung tissue of asthmatic *Sirt1^fl/fl^* mice compared with those in control groups (Figure 1B, 1C). The expression of SIRT1 in the lung tissue of model mice was further confirmed by WB and RT-PCR (Figure 1D, 1E). We next sought to identify specific cell types related to abnormal expression of SIRT1. We found that SIRT1 was mainly expressed in lung macrophages of asthmatic *Sirt1^fl/fl^* mice (Figure 1F, 1G), and similar results were found in the bronchoalveolar lavage (BALF) samples (Figure 1H, 1I).

**Figure 2:**
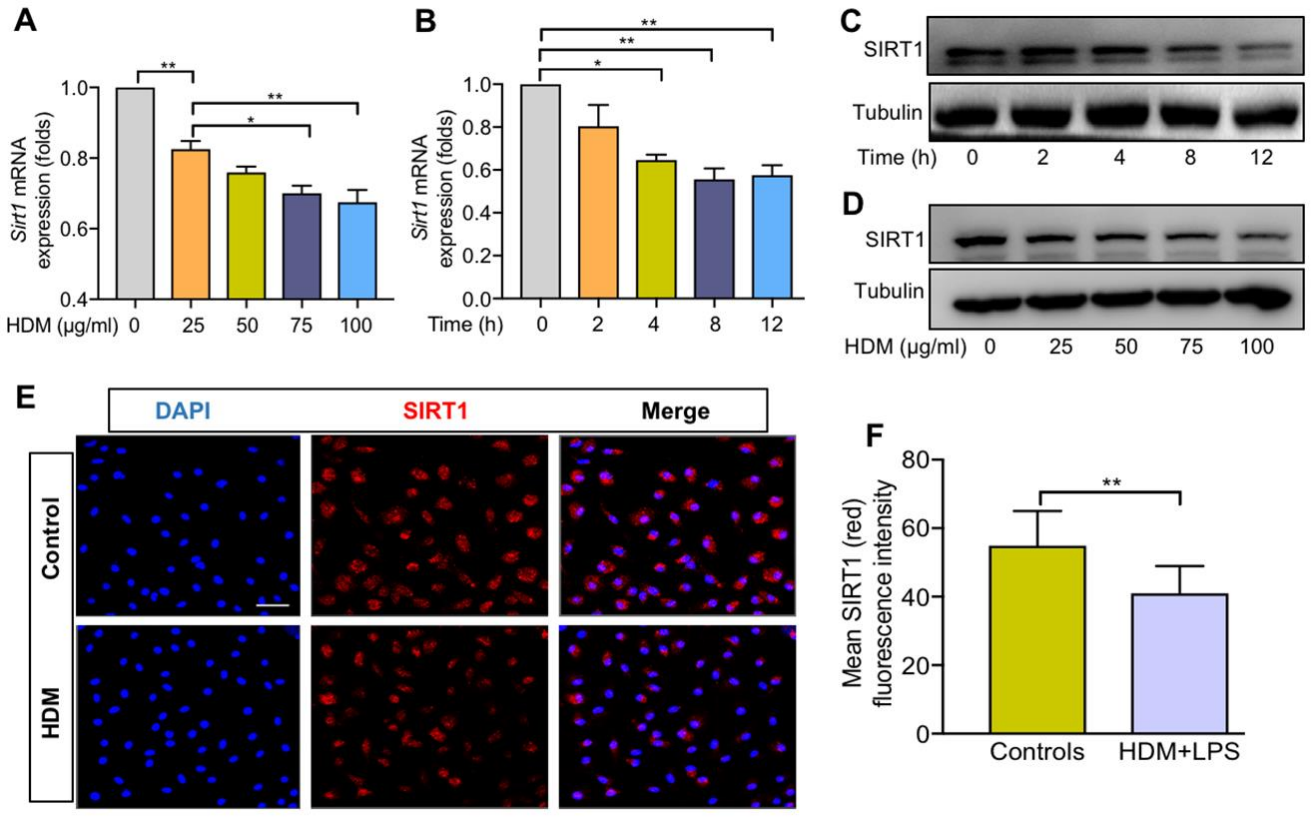
**SIRT1 expression was decreased in BMDMs exposed to HDM.** Dose–dependent expression of SIRT1 in bone marrow–derived macrophages (BMDMs) stimulated with HDM for 24 h and was analyzed using RT-PCR (**A**) and WB (**C**); Time–dependent expression of SIRT1 in BMDMs treated with HDM at 100 μg/ml and was analyzed using RT-PCR (**B**) and Western blot (**D**); BMDMs treated with HDM at 100 μg/ml for 24 h. Representative immunofluorescence images of SIRT1 (red) expression in BMDMs (**E**, Scale bar, 50 μm) and semiquantification of immunofluorescence images was done using Image Pro 6.1 software (**F**). Data are presented as Mean ± SEM of three independent experiments. *P<0.05, **P<0.01 and ***P<0.001.

To further explore the role of SIRT1 in lung macrophages, we generated BMDMs from *Sirt1^fl/fl^* mice. Consistent with the above findings, the expression of *Sirt1* gene was significantly reduced in HDM-induced BMDMs in a dose- and time-dependent manner (Figure 2A, 2B). SIRT1 protein levels in BMDMs were confirmed by using WB and immunofluorescence (IF) analysis (Figure 2C–2E). Taken together, these findings suggested that macrophages are involved in SIRT1-mediated airway allergic inflammation.

### *Sirt1*-deficient BMDMs increase cytokine secretion following HDM exposure

**Figure 3:**
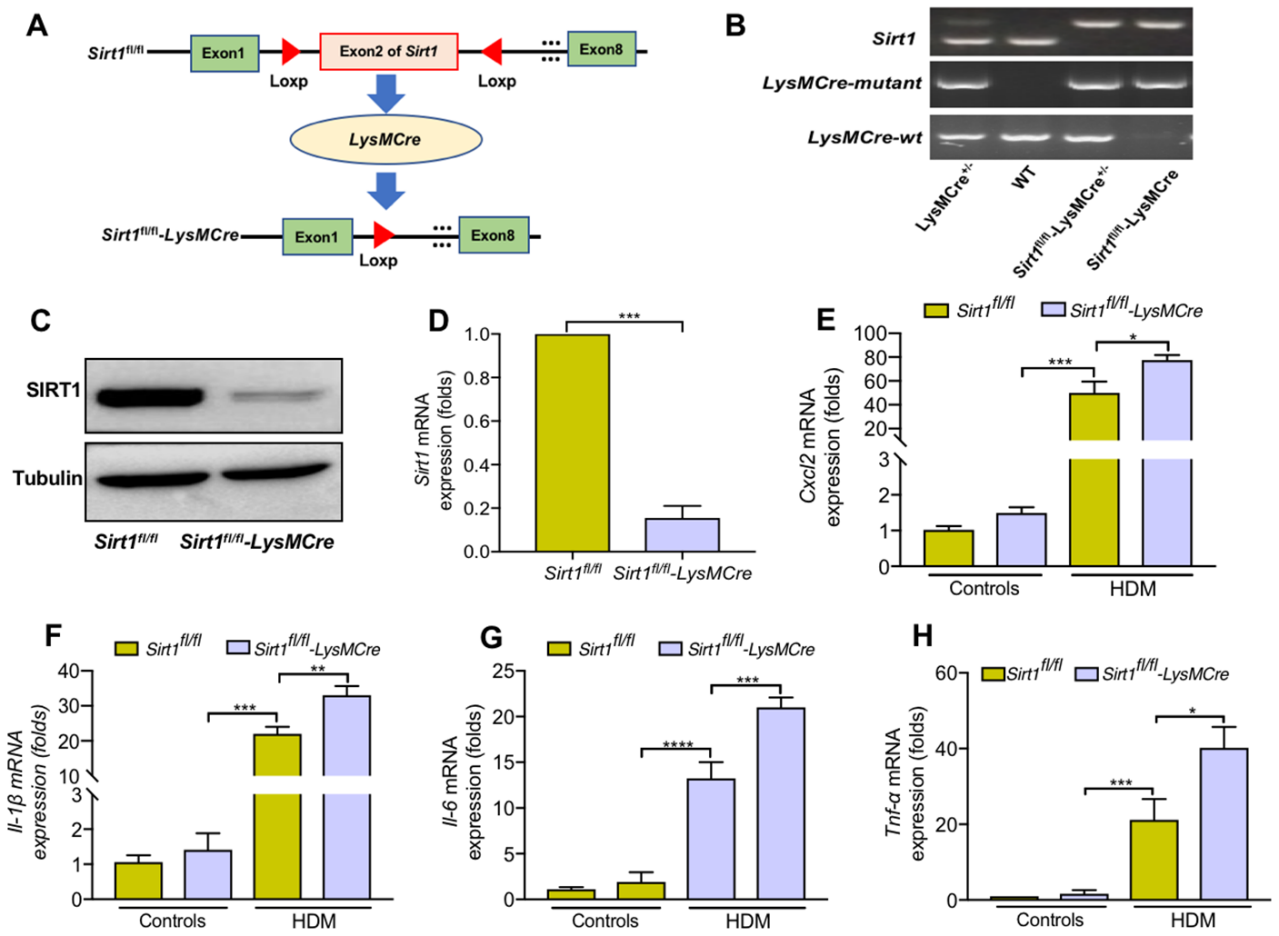
***Sirt1*-deficient BMDMs enhanced cytokine secretion when treated with HDM.** (**A**) Schematic map of the generation of *Sirt1^fl/fl^-LysMCre* mice. (**B**) Genotyping was performed by PCR using mouse tail genomic DNA. (**C**, **D**) Genotyping was assessed in BMDMs from *Sirt1^fl/fl^* and *Sirt1^fl/fl^-LysMCre* mice using Western blot and RT-PCR analysis. (**E**–**H**) BMDMs were treated with HDM at 100 μg/ml for 24 h to measure the levels of *Cxcl2, Il-1β, Il-6, and Tnf-α*. Data are presented as Mean ± SEM of three independent experiments. *P<0.05, **P<0.01, ***P<0.001 and ****P<0.0001.

We next generated mice with myeloid conditional deletion of *Sirt1* (mainly in macrophages) (*Sirt1^fl/fl^*-*LysMcre*, Figure 3A). Representative genotyping results as shown in Figure 3B. The genotyping results were further determined by WB and RT-PCR analysis (Figure 3C, 3D). *Sirt1^fl/fl^-LysMCre* mice had a normal weight and lifespan. No pathological changes were observed in the lungs of *Sirt1^fl/fl^-LysMCre* mice (Supplementary Figure 1A). Moreover, *Sirt1^fl/fl^-LysMCre* mice did not show defects in the development and differentiation of immune cells (Supplementary Figure 1B–1F).

We generated BMDMs from *Sirt1^fl/fl^-LysMCre* and *Sirt1^fl/fl^* mice, and found that *Il-6, Tnf-α, Cxcl2, and Il-1β* expression were significantly increased in *Sirt1*-deficient BMDMs compared with the control group (Figure 3E–3H). Collectively, these findings imply that loss of SIRT1 in macrophages enhanced inflammatory cytokine response.

### *Sirt1^fl/fl^-LysMCre* mice display exacerbated airway inflammation

**Figure 4:**
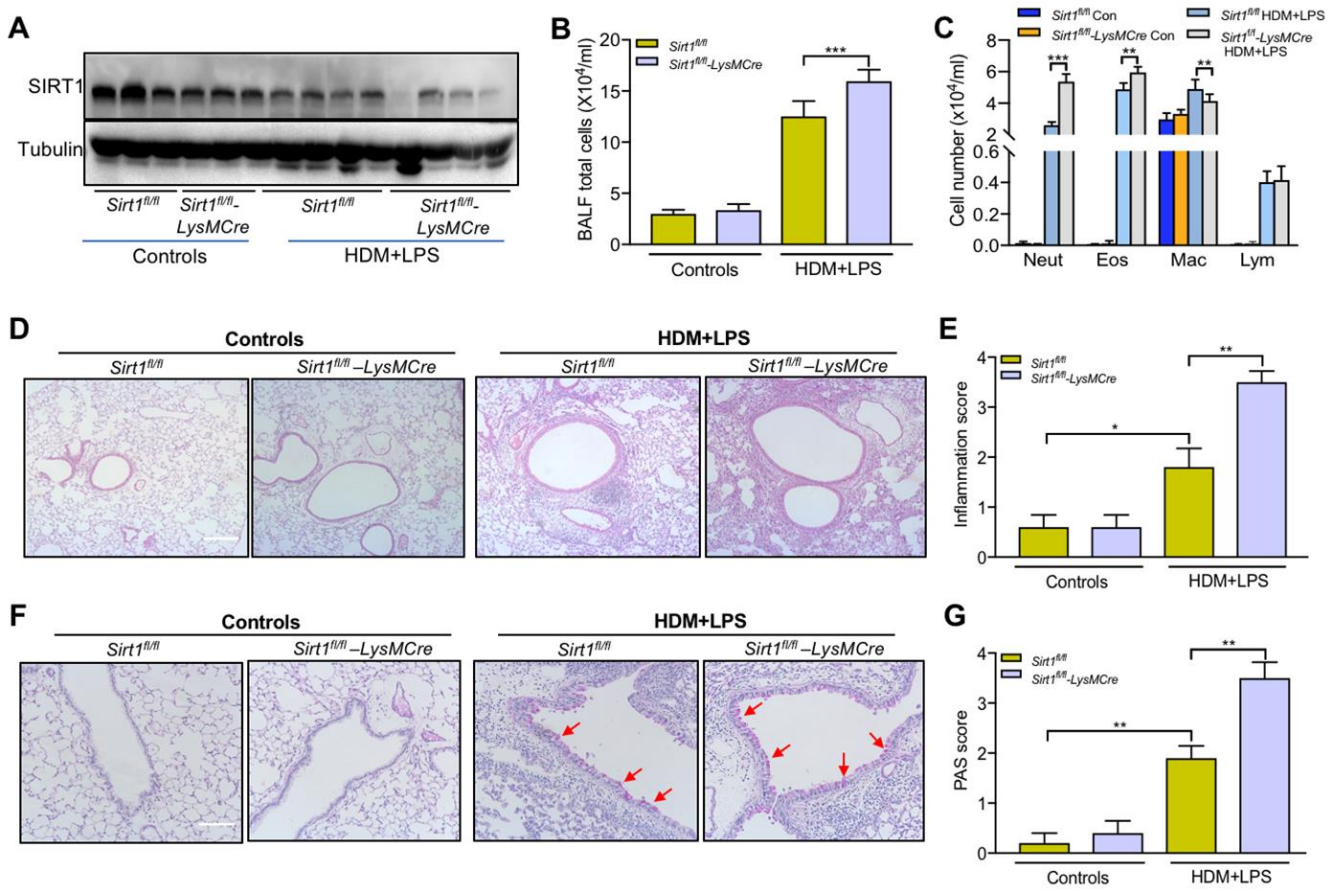
**Impairing of SIRT1 exacerbates airway inflammation following allergen exposure.** *Sirt1^fl/fl^-LysMCre* mice and *Sirt1^fl/fl^* littermates were established allergic asthma model as described in the methods section (n = 5-8 for each group). (**A**) SIRT1 expression in lung tissues of *Sirt1^fl/fl^* and *Sirt1^fl/fl^-LysMCre* mice were assessed using Western blot; (**B**, **C**) Total cells and differential cell counts in BALF were measured; (**D**) Representative photomicrographs of lung inflammation expression are shown (Scale bar, 100 μm); (**E**) Semiquantification of inflammation expression in the lungs were preformed using Image Pro 6.1 software; (**F**) Representative photomicrographs of mucus production are shown (red arrows) (Scale bar, 100 μm); (**G**) Semiquantification of mucus production in the lungs were preformed using Image Pro 6.1 software. Data are presented as Mean ± SEM of three independent experiments. *P<0.05, **P<0.01 and ***P<0.001.

To further elucidate the role of SIRT1 in allergic airway inflammation *in vivo,* we established allergic asthma model using *Sirt1^fl/fl^* littermates and *Sirt1^fl/fl^-LysMCre* mice, as described in Figure 1A Compared with asthmatic *Sirt1^fl/fl^* mice, SIRT1 expression in lung tissue was significantly decreased in asthmatic *Sirt1^fl/fl^-LysMCre* mice (Figure 4A).

**Figure 5:**
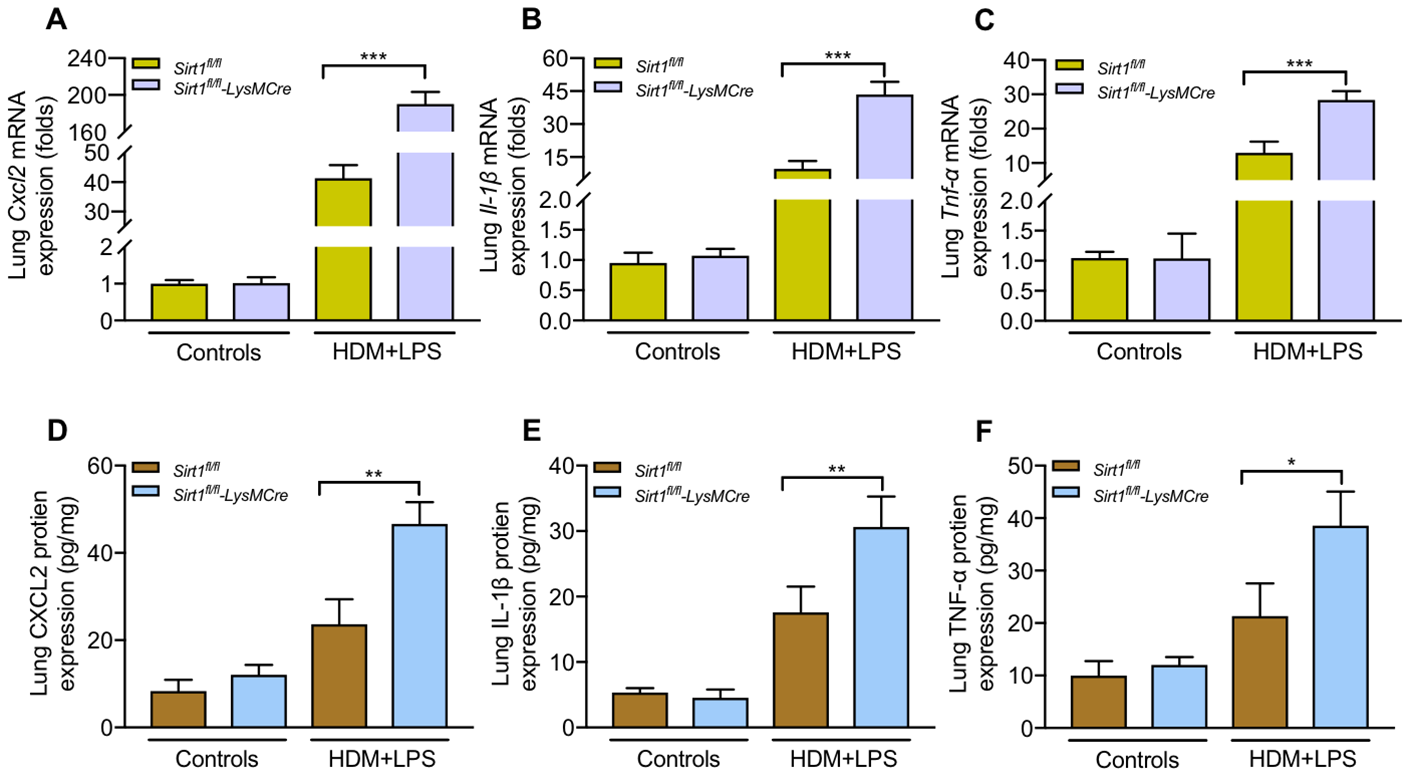
***Sirt1*-deficient exacerbates inflammatory cytokines production following allergen exposure.** *Sirt1^fl/fl^-LysMCre* mice and *Sirt1^fl/fl^* littermates were established allergic asthma model (n = 5-8 mice per group per study). Lungs were isolated 1 day after the last challenge. (**A**–**C**) Expression of the mRNA levels of inflammatory cytokines in the lung homogenate were analyzed by RT-PCR; (**D**–**F**) Protein levels of inflammatory cytokines in the lung homogenate were measured by ELISA. Data are representative of three independent experiments with similar results. Data are presented as Mean ± SEM of three independent experiments. *P<0.05, **P<0.01 and ***P<0.001.

The total BALF cells, eosinophils and neutrophils were significantly increased in BALF of asthmatic *Sirt1^fl/fl^-LysMCre* mice compared with those in asthmatic *Sirt1^fl/fl^* mice (Figure 4B, 4C). Moreover, airway inflammation and mucus secretion were significantly increased in *Sirt1^fl/fl^-LysMCre* mice exposed to the allergen (Figure 4D–4G). Compared with the *Sirt1^fl/fl^* mice, both mRNA and protein expression of TNF-α, CXCL2, and IL-1β in the lung tissue were significantly enhanced in allergen-exposed *Sirt1^fl/fl^-LysMCre* mice (Figure 5).

### SIRT1 suppresses airway inflammation partially through inhibition of ERK/p38 MAPK pathway activation

**Figure 6:**
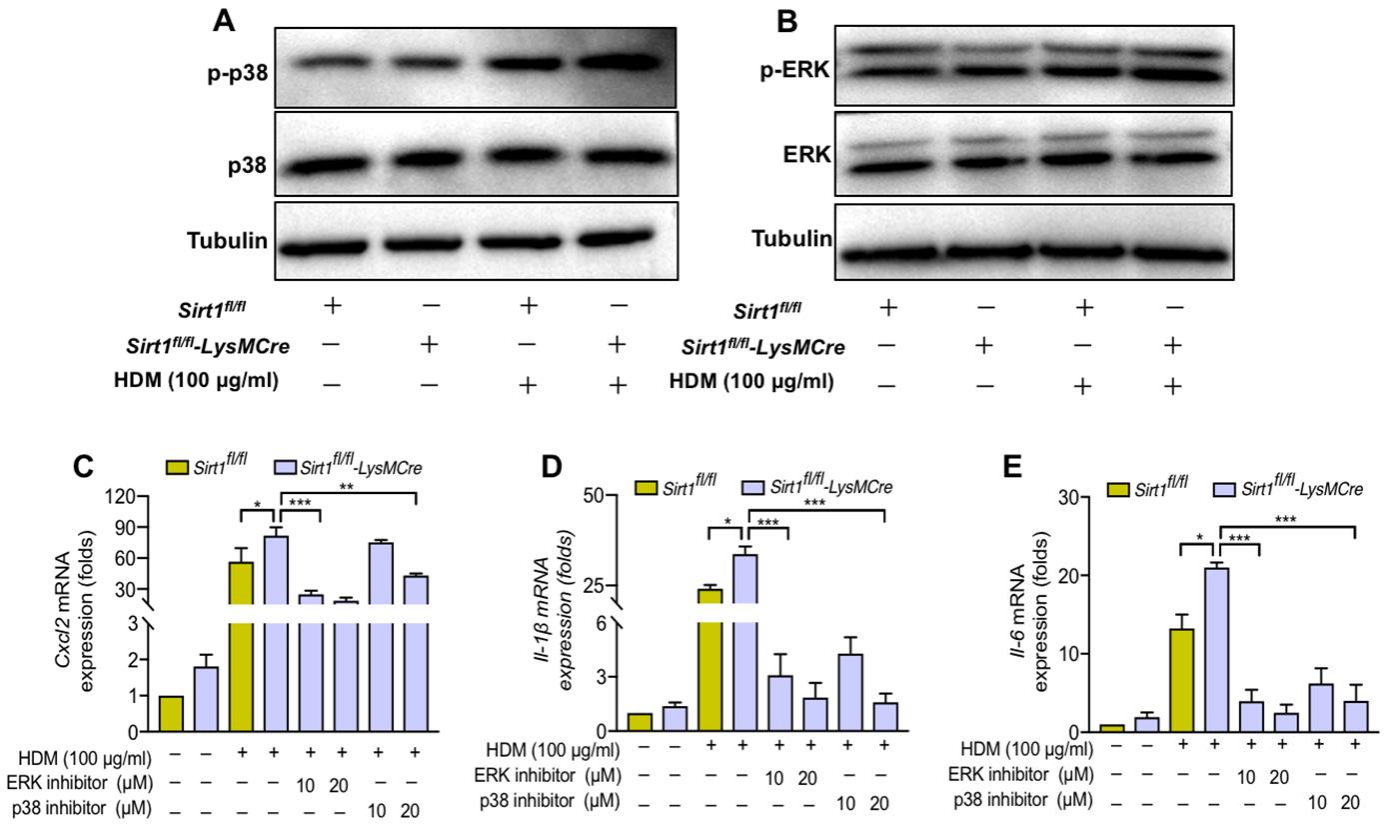
**SIRT1 suppresses airway inflammation partially through inhibition of ERK/p38 MAPK pathway activation.** (**A**, **B**) *Sirt1^fl/fl^* and *Sirt1^fl/fl^-LysMCre* BMDMs were treated with HDM, the expression of p-p38 and p-ERK was assessed by using Western blot analysis; (**C**–**E**) p38 inhibitor SB203580 and ERK inhibitor U0126 were added in HDM-exposed BMDMs and BMDMs were collected after 24 h. The levels of *Cxcl2, Il-1β,* and *Il6* were analyzed by RT-PCR. Data are representative of three independent studies. Results are expressed as mean ± SEM. *P<0.05, **P<0.01 and ***P<0.001.

Finally, we assessed the underlying pathways that mediate SIRT1 in regulation of airway inflammatory in macrophages. The mitogen-activated protein kinase (MAPK) pathways is involved in airway inflammation. Therefore, we sought to determine whether MAPK pathways mediate the role of SIRT1 in regulation of inflammatory responses. We found that the phosphorylation of p38 and ERK was increased in HDM-induced BMDMs (Supplementary Figure 2). Moreover, the p-p38 and p-ERK expression in *Sirt1^fl/fl^-LysMCre* mice were further aggravated compared with the *Sirt1^fl/fl^* mice (Figure 6A, 6B).

To further validate the role of ERK/p38 MAPK pathway in SIRT1 mediated- airway inflammation in asthma, we used the p38 inhibitor SB203580 and the ERK inhibitor U0126 to examine whether blocking ERK/p38 MAPK activation could reverse the amplified cytokine production caused by SIRT1 deficiency. We found that the levels of inflammatory cytokines were significantly decreased in SIRT1-deficient BMDMs treated with SB203580 and U0126. (Figure 6C–6E).

These results suggested that SIRT1 deficiency enhanced ERK/p38 activation and cytokine production in macrophages.

## DISCUSSION

The present study showed that SIRT1 expression was decreased in HDM-induced BMDMs while *Sirt1*-deficient BMDMs increase cytokine secretion when treated with HDM. Moreover, mice with myeloid cells specific knockdown of SIRT1, display exacerbated airway inflammation. ERK/p38 MAPK pathway is the possible underlying mechanism of SIRT1 mediated suppression of HDM-induced airway inflammation in macrophages. Our study suggested that activation of SIRT1 in macrophages may represent therapeutic strategy for asthma.

Sirtuins regulate diverse biological functions such as stress resistance, metabolism, and aging. SIRT1 plays a critical role in inflammatory diseases by regulating transcription factors, airway inflammation, apoptosis and senescence [9]. However, the roles of SIRT1 in regulating macrophage activation in the pathogenesis of asthma remain unclear [5]. Macrophages from healthy subjects produce lesser IL-10 and co-stimulatory molecules (CD80 and CD86) than asthmatic patients [10]. A study revealed that macrophages from unsensitized mice provided protection against asthmatic symptoms [11]. In contrast, other studies demonstrated that macrophages play a minor role in airway allergic inflammation [12, 13]. The disparity in these studies may be attributed to the different macrophage studied. Our study focused on the function of macrophages in regulation of HDM-induced airway inflammation.

SIRT1 is involved in regulation of the inflammatory response in macrophages [14, 15]. It has been reported that SIRT1 induced macrophage polarization and attenuated monosodium urate crystal-induced inflammation [16]. Myeloid-specific deletion of SIRT1 enhanced obesity-induced inflammation [17]. Similarly, mouse macrophage specific knockout of SIRT1 exacerbated abdominal aortic aneurysm formation [18]. In the context of insulin resistance, macrophage SIRT1 may play a beneficial role in regulating glucose homeostasis [19]. However, the mechanisms of macrophage SIRT1 in airway allergic inflammation remain largely unknown. Here, we found that SIRT1 expression is reduced in HDM-induced *Sirt1^fl/fl^* BMDMs. HDM exposure significantly enhanced cytokine production in *Sirt1^fl/fl^* BMDMs, and BMDMs deficient in *Sirt1* further increased cytokine production. Consistent with *in vitro* experiments, *Sirt1^fl/fl^-LysMCre* mice showed substantially enhanced airway inflammation, cytokine production, and mucus production compared with allergen exposed *Sirt1^fl/fl^-LysMCre* mice. Further, we found that SIRT1 suppresses airway inflammation at least partially through inhibition of ERK/p38 MAPK pathway activation. Collectively, these data suggest that downregulation of SIRT1 could be a necessary mechanism by which allergic airway inflammation occurs.

Our study does have some limitations. First, macrophages are polarized towards the M2 phenotype in asthma. Whether SIRT1 mediates the M2-type AM polarization and involves in the pathogenesis of asthma remain to be further elucidated. Second, since SIRT1 is an NAD-dependent deacetylase, SIRT1's deacetylation activity via epigenetics is one of its most important functions. Thus, the epigenetic role of SIRT1 in macrophages of asthma also needs to be further investigated.

In conclusion, our study reveals that SIRT1 suppresses the allergic airway inflammation in macrophages, and suggested that activation of SIRT1 in macrophages might be a strategy for treating the allergic airway inflammation.

## MATERIALS AND METHODS

### Animal studies

The *LysMcre* mice were a generous gift from Dr. G. Feng (University of California at San Diego, CA, USA). *Sirt1^fl/fl^* mice were purchased from GemPharmatech Co., Ltd., and. Myeloid cell-specific SIRT1conditional knockout mice (*Sirt1^fl/fl^-LysMCre*) were obtained by crossing *Sirt1^fl/fl^* mice with the *LysMCre* mice. Sequence of primers for *LysMCre* and *Sirt1* genes are listed in Table 1. All experimental procedures were approved by the Guangdong Medical University’s Animal Ethical Committee.

**Table 1:** Sequence of primers were used in this study.

**Genes**	**Forward**	**Reverse**
*Sirt1 (m)*	5ʹ-CACTGTAACTGGGGGCAACT-3’	5ʹ-CACTTCTTGTCAGCGTCGAA-3’
*Cxcl-2(m)*	5ʹ-TGTCCCTCAACGGAAGAACC-3’	5ʹ-CTCAGACAGCGAGGCACATC-3’
*Il-1β(m)*	5ʹ-GCAACTGTTCCTGAACTCAACT-3’	5ʹ-ATCTTTTGGGGTCCGTCAACT-3’
*Tnf-α(m)*	5ʹ-GACGTGGAACTGGCAGAAGAG-3’	5ʹ-TTGGTGGTTTGTGAGTGTGAG-3’
*β-actin(m)*	5ʹ-AGTGTGACGTTGACATCCGT-3’	5ʹ-GCAGCTCAGTAACAGTCCGC-3’
*LysMCre-WT(m)*	5ʹ-TTACAGTCGGCCAGGCTGAC-3’	
*LysMCre-Mutant(m)*	5ʹ-CCCAG AAATGCCAGATTACG-3’	
*LysMCre-Common(m)*	5ʹ-CTTGGGCTGCCAGAATTTCTC-3’	
*Sirt1 gene deletion(m)*	oIMR7909: 5ʹ-GGTTGACTTAGGTCTTGTCTG-3’	
	oIMR7912: 5ʹ-CGTCCCTTGTAATGTTTCCC-3’	

### BMDMs

BMDMs were isolated and cultured as described previously [20]. Briefly, BM cells were collected from six- to eight week-old male mice. Red blood cells (RBCs) Lysing Buffer (Tbdscience.com) was used to remove red blood cells. The remaining cells were incubated in DMEM containing antibiotics, FBS (10%), and 10 ng/ml recombinant mouse M-CSF (Novoprotein, Catalog #0331488) for 7 days to promote differentiate bone marrow-derived macrophages.

### Allergic asthma mouse model

Allergic asthma mouse model was established according to previous studies [21–23]. Briefly, six- to eight-week-old mice were sensitized by intratracheal instillation of 5 μg HDM (Greer Laboratories) plus 3 μg LPS (Sigma) in 50 μl normal saline (NS) or with 50 μl NS on days 0, 1, and 2. On days 15 and 16 after the initial sensitization, the mice were challenged with 2.5 μg HDM plus 1.5 μg LPS in 50 μl NS or with 50 μl NS using intratracheal instillation. The mice were sacrificed under anesthesia 24 h after the last challenge. Bronchoalveolar lavage fluid (BALF) was conducted according to our previous study [2]. Cytospin slides were performed using the Wright-Giemsa staining method. Hematoxylin/eosin (HE) staining sections and mucus production of goblet cells were assessed using a semiquantitative scoring system as previously described [17]. RT-PCR analysis was preformed according to our previous study [2]. The sequence of primers is listed in Table 1.

### Immunohistochemistry (IHC)

The slides were detected with a rabbit polyclonal antibody against SIRT1 (Abcam). Semiquantitative evaluation of SIRT1 positive cells was analyzed with the Image Pro 6.1 software following previously described methods [2].

### Western blot

Lysates from BMDMs and lung tissues were separated by SDS-PAGE and immunoblotted as previously described methods [2], using antibodies against the following proteins: SIRT1 (Abcam), Tubuin (Beyotime), p-ERK1/2, ERK, p38, and p-p38 (CST).

### ELISA

The expression of TNF-α, CXCL2, and IL-1β in lung homogenate were detected by ELISA kits (eBioscience) following the manufacturer’s recommendation.

### Flow cytometry

Flow cytometry was performed on a Fortessa (BD Biosciences) as previously described methods [2]. Anti-mouse CD19-Percp, CD8-PE-Cy7, F4/80-APC, CD3-APC-Cy7, CD11c-PE, CD4-FITC, and CD49b-PE were purchased from Biolegend.

### Immunofluorescence (IF)

The expression of SIRT1 in alveolar macrophages (AMs) and BMDM cells was detected by immunofluorescence as previously described methods [2]. DAPI was used for nucleus staining.

### Statistical analysis

Results are described as mean with SEM. All calculations and the graph were conducted using GraphPad Prism (San Diego, CA, version 8.0). Mann-Whitney U-test or Student's t test was used to compare differences between two groups. For comparisons between more than two groups, one-way ANOVA was applied. A value of P less than 0.05 was considered statistically significant.

## Supplementary Material

Supplementary Figures
