# Supplementary material for: Myeloid-specific SIRT1 deletion exacerbates airway inflammatory response in a mouse model of allergic asthma
Source: Aging (Albany NY). 2021 Jun 7;13(11):15479–90. doi: 10.18632/aging.203104 (PMC8221322; doi:10.18632/aging.203104)
Supplement: Supplementary Figures [file aging-13-203104-s001.pdf]

SUPPLEMENTARY FIGURES

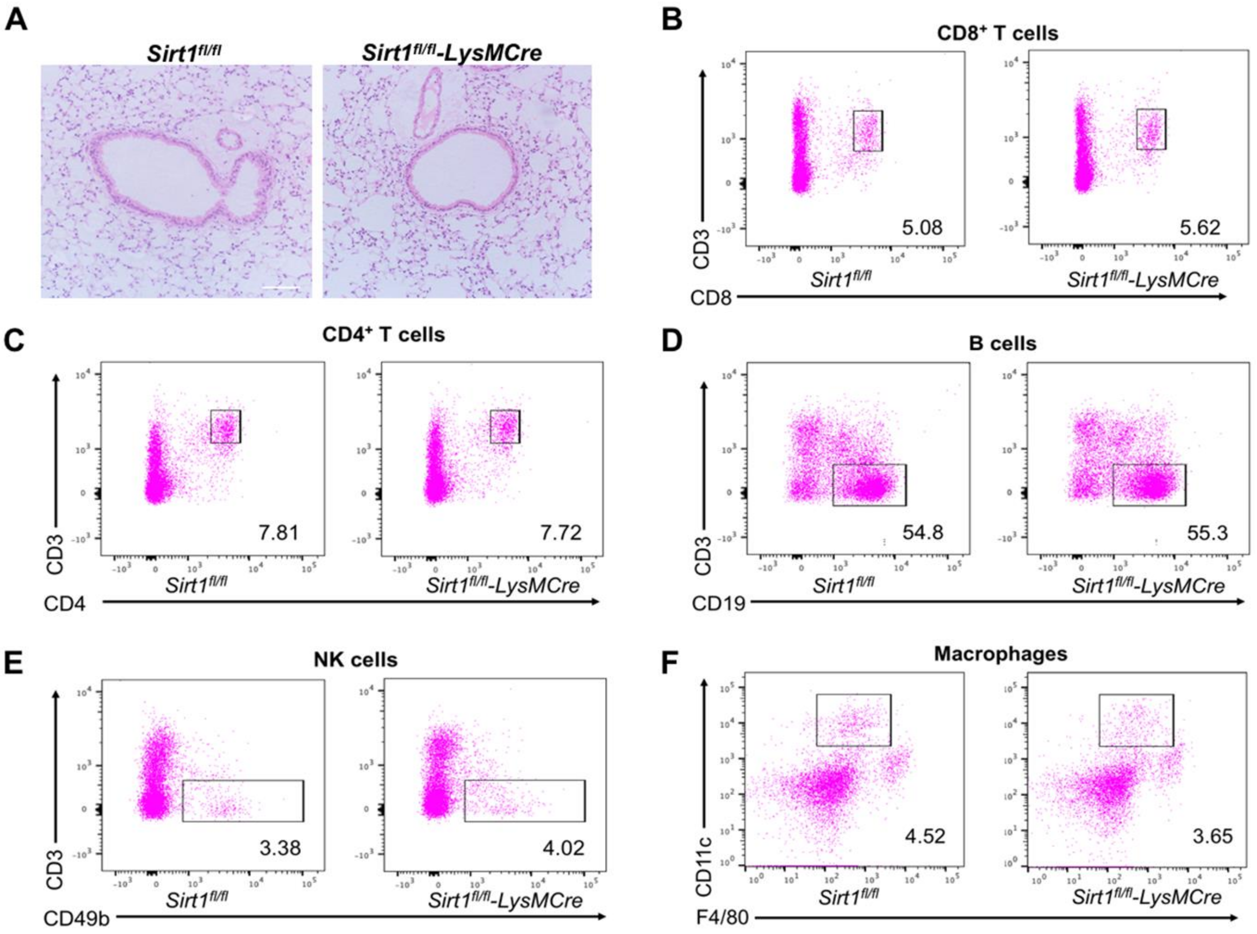

Supplementary Figure 1. *Sirt1* deficiency has no influence on cell development and differentiation *in vivo*. (**A**) The lung sections were histologic analyzed using HE staining to visualize inflammatory cell recruitment (Scale bar, 100 μm). (**B**–**F**) The $CD45^+CD3^+$ cells in the splenocytes of *Sirt1*$^{fl/fl}$ and *Sirt1*$^{fl/fl}$*-LysMCre* mice were gated for further analysis of $CD8^+$ T cells, $CD4^+$ T cells, B cells, and NK cells. The $CD45^+CD11c^+F4/80^+$cells in the splenocytes of *Sirt1*$^{fl/fl}$ and *Sirt1*$^{fl/fl}$*-LysMCre* mice were gated for macrophages. Representative flow cytometer of $CD8^+$ T cells, $CD4^+$ T cells, B cells, and NK cells, and macrophages expression are shown.

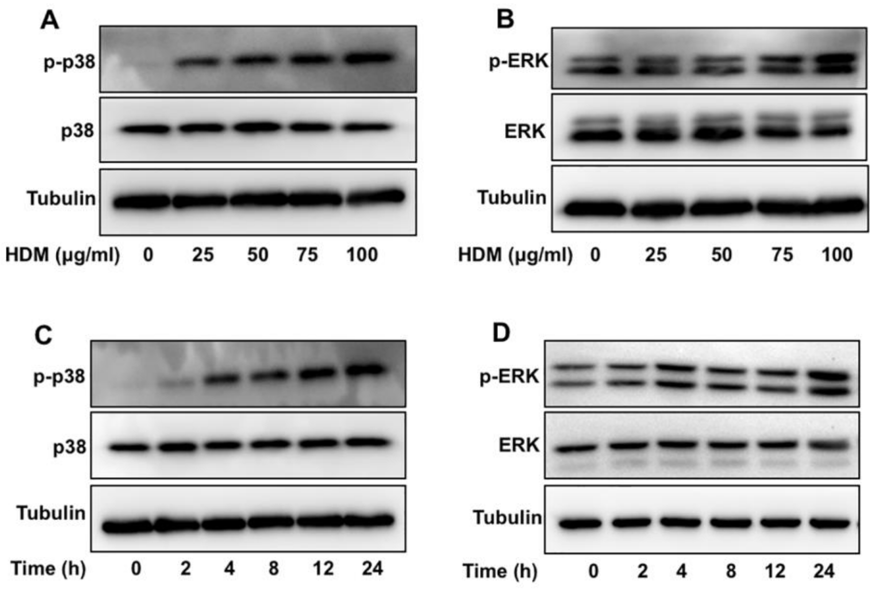

Supplementary Figure 2. HDM-induced inflammation response in BMDMs via MAPK pathways. BMDMs were treated with HDM, and the protein levels of MAPK pathways were measured by Western blot. (**A, B**) HDM activated p38 and ERK phosphorylation in a dose-dependent manner (0, 25, 50, 75 and 100 µg/ml for 24 h). (**C, D**) Stimulation of BMDMs with100 µg/ml of HDM induced phosphorylation of p38 and ERK in a time-dependent manner (0, 2, 4, 8, 12, 24 h).
